# Comparative Transcriptome Analysis of Two Oysters, *Crassostrea gigas* and *Crassostrea hongkongensis* Provides Insights into Adaptation to Hypo-Osmotic Conditions

**DOI:** 10.1371/journal.pone.0111915

**Published:** 2014-11-04

**Authors:** Xuelin Zhao, Hong Yu, Lingfeng Kong, Shikai Liu, Qi Li

**Affiliations:** Key Laboratory of Mariculture, Ministry of Education, Ocean University of China, Qingdao, 266003, China; Sars International Centre for Marine Molecular Biology, Norway

## Abstract

Environmental salinity creates a key barrier to limit the distribution of most aquatic organisms. Adaptation to osmotic fluctuation is believed to be a factor facilitating species diversification. Adaptive evolution often involves beneficial mutations at more than one locus. Bivalves hold great interest, with numerous species living in waters, as osmoconformers, who maintain the osmotic pressure balance mostly by free amino acids. In this study, 107,076,589 reads from two groups of *Crassostrea hongkongensis* were produced and the assembled into 130,629 contigs. Transcripts putatively involved in stress-response, innate immunity and cell processes were identified according to Gene ontology and KEGG pathway analyses. Comparing with the transcriptome of *C. gigas* to characterize the diversity of transcripts between species with osmotic divergence, we identified 182,806 high-quality single nucleotide polymorphisms (SNPs) for *C. hongkongensis*, and 196,779 SNPs for *C. gigas*. Comparison of 11,602 pairs of putative orthologs allowed for identification of 14 protein-coding genes that experienced strong positive selection (Ka/Ks>1). In addition, 45 genes that may show signs of moderate positive selection (1≥Ka/Ks>0.5) were also identified. Based on Ks ratios and divergence time between the two species published previously, we estimated a neutral transcriptome-wide substitution mutation rate of 1.39×10^−9^ per site per year. Several genes were differentially expressed across the control and treated groups of each species. This is the first time to sequence the transcriptome of *C. hongkongensis* and provide the most comprehensive transcriptomic resource available for it. The increasing amount of transcriptome data on *Crassostrea* provides an excellent resource for phylogenetic analysis. A large number of SNPs identified in this work are expected to provide valuable resources for future marker and genotyping assay development. The analysis of natural selection provides an innovative view on the adaptation within species and sets the basis for future genetic and evolutionary studies.

## Introduction

Mollusca is one of the most species phyla of invertebrates and possess a global-scale increase in species richness from the poles to the equator [1]. As its main class, bivalves can adapt to many kinds of hostile environments, such as intertidal zones with drastic fluctuations of environmental factors and deep sea vents with extreme cold, high pressure and darkness [2], [3]. Bivalves, as one of the most important inhabitants of water bodies, take part in the maintenance of ecosystem stability and biodiversity [4], [5]. However, how bivalves possess the adaptations in response to the complex and changeable living conditions, such as salinity, temperature and pH, is still a mystery on a genetic basis.

The advances of adaptation studies would facilitate progress in many fields of biology [6], therefore, elucidating the process of adaptation and understanding its genetic basis are the main objectives of evolutionary biology [7]. Despite tremendous advances in genetic studies, a link between adaptive phenotypes and genotypes has been made for only a small number of traits in an even smaller number of organisms [8]–[10]. These studies mainly focused on model organisms and a few candidate genes, while understanding of the molecular basis of adaptation in non-model species remains largely unknown.

Recent development of next generation sequencing (NGS) technology and bioinformatic tools enable us to analyze massive sequence data efficiently and cost-effectively [11], [12]. The NGS based approaches hold great potential to expand genomic resources for any non-model organism and allows large-scale comparative analysis with genomes or transcriptomes [13]–[18]. Transcriptome or genome sequencing have been conducted for various marine bivalves, such as clam [19], mussels [2], [20], and oysters [15], [21]. In addition to candidate gene discovery from massive sequencing data, many studies have demonstrated that this is an efficient way to discover genetic variations [22]–[24], perform transcriptome profiling [25]–[27], and identify adaptive genes [28], [29] to provide important insights into the process of adaptive evolution.

Oysters (*Crassostrea* sp.) are one of the most important species in bivalves for their economic importance as an aquaculture species, global distribution and wide use for research. They are benthic, sessile filter-feeders, and are widely distributed in the world estuaries and coastal zones [30]. There are mainly five *Crassostrea* species along China’s coasts [31]. Of which, *C. gigas* and *C. hongkongensis* contribute to different fauna assemblages. The oyster *C. gigas* is a eurythermal and euryhaline species which widely spreads around the world and inhabits the northern and southern intertidal zones in China. The optimal salinity for *C. gigas* is above 20‰ [32]. The oyster *C. hongkongensis* lives under estuarine conditions around southern China [33], and grows in salinity from 10‰ to 20‰ [34]. Due to the differences in salinity adaptation, *C. gigas* and *C. hongkongensis* provide an excellent model system to study how bivalves adapt to hypo-osmotic conditions.

In this study, we conducted transcriptome sequencing of *C. hongkongensis* gill tissues using the Illumina sequencing platform. By revisiting the transcriptomic data of *C. gigas* that has been reported previously [27], we are aiming to perform a genome-wide analysis for genes that may be involved in adaptation to hypo-osmotic environments.

## Results and Discussion

### Sequencing and assembly

**Figure 1:**
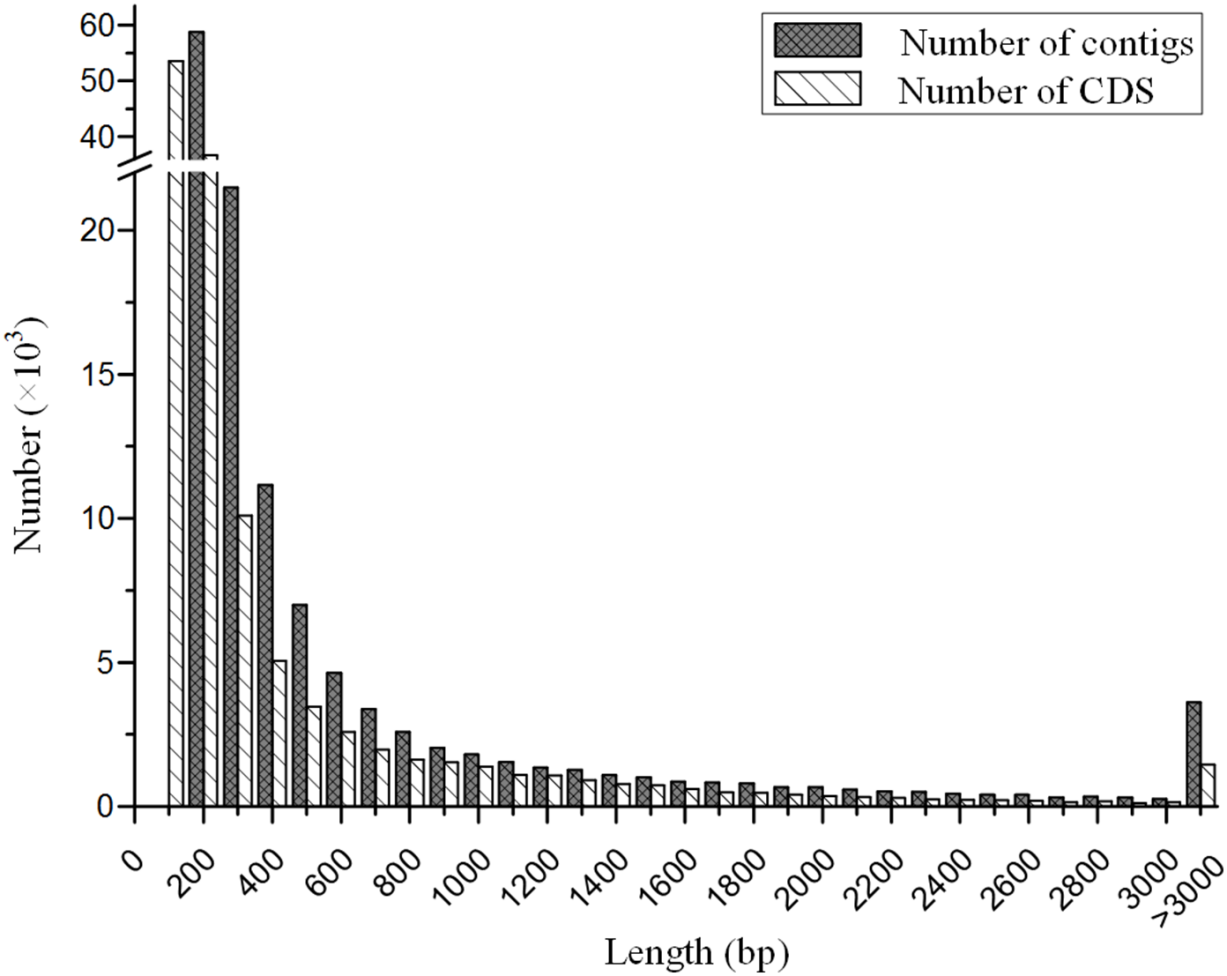
The length distribution of contigs and coding sequences (CDSs) of *C. hongkongensis*. Contigs were generated from *de* *novo* assembly of Illumina sequencing reads. The minimum length of contigs was limited to 200 bp and that of CDSs was limited to 100 bp.

The two cDNA libraries prepared used RNA from the two groups of *C. hongkongensis*, which included one group accumulated in filtered seawater with optimal salinity (HC group) and another group accumulated in that with low salinity (HT group). A total of 62,643,682 sequence reads from HC group, and 61,096,062 sequence reads from HT group were generated by Illumina sequencing, respectively. These reads have been deposited in the NCBI GEO database with the accession number of GSE51157. After trimming, a total of 54,032,237 and 53,044,352 clean reads were obtained, respectively (Table 1). Assembly of these reads generated 130,629 unique transcript sequences, with the lengths ranging from 201 to 21,597 bp and an average length of 645 bp (Figure 1). Similarly, we performed the *de* *novo* assembly with the transcriptomic data from *C. gigas* [27]. Apparently, the lower throughput sequencing in *C. gigas* resulted in the assembly of shorter contiguous sequences as compared to *C. hongkongensis*. The *de* *novo* assembly generated 300,382 unique transcript sequences for *C. gigas*, with the average length of 419 bp (Table 1).

**Table 1:** Summary of the transcriptome assembly for *C. hongkongensis* and *C. gigas.*

	*C. hongkongensis*		*C. gigas*	
	HT	HC	PT	PC
Raw data	61,096,062	62,643,682	13 719 859	15 354 006
Clean data	53 044 352	54 032 237	13 573 056	15 237 315
Read length (bp)	100	100	92	92
N50 length of assembly (bp)	1117		831	
Mean length of assembly (bp)	645		419	
Total number of transcripts	130,629		300,382	
Number of putative orthologs	11,602			

Obviously, the assembled transcript sequences drastically outnumbered the protein coding genes in both species, and a large portion of sequences were short with the length of 200–300 bp (Figure 1). One of the reason could be that different isoforms of same genes produced from alternative splicing processes were assembled into separate transcript sequences. However, we should acknowledge that a large proportion of transcripts were not assembled into full-length sequences due to insufficient sequencing coverage. This was supported by the observation in *C. gigas* where the assembly with 28.8 million reads was much poorer than that in *C. hongkongensis* with over 107 million reads (Table 1). Additionally, the high levels of heterozygosity and allelic variations in oysters would also make the *de* *novo* assembly difficult, resulting in short fragmented sequences.

### Gene annotation

The annotation was conducted first based on the *ab initio* prediction of protein coding sequences. Of the 130,629 assembled transcript sequences from *C. hongkongensis*, 128,481 sequences were predicted to contain CDSs with the minimum length of 100 bp. The lengths of CDSs ranged from 102 bp to 21,594 bp with an average length of 423 bp (Figure 1). Then, the predicted protein-coding sequences were searched against the public protein databases with an E-value cut-off of 1e–5 using Blastp. Of the 128,481 transcript sequences with CDSs, a total of 41,776 were annotated with inferred gene identities and 23,916 were remained after removing redundancy. Among them, 15,626 sequences (65.3%) were annotated based on the genome of *C. gigas* [15].

Taken together, only ∼32% (41 776/130 629) transcript sequences with unique putative CDSs were successfully annotated in this study. The annotation efficiency was comparable with those reported in other *de* *novo* transcriptome sequencing studies for non-model organisms [2], [22], [35]–[37]. This could be largely due to the incomplete assembly and the lack of genomic information in public databases for bivalves.

### Gene ontology and pathway analysis

**Figure 2:**
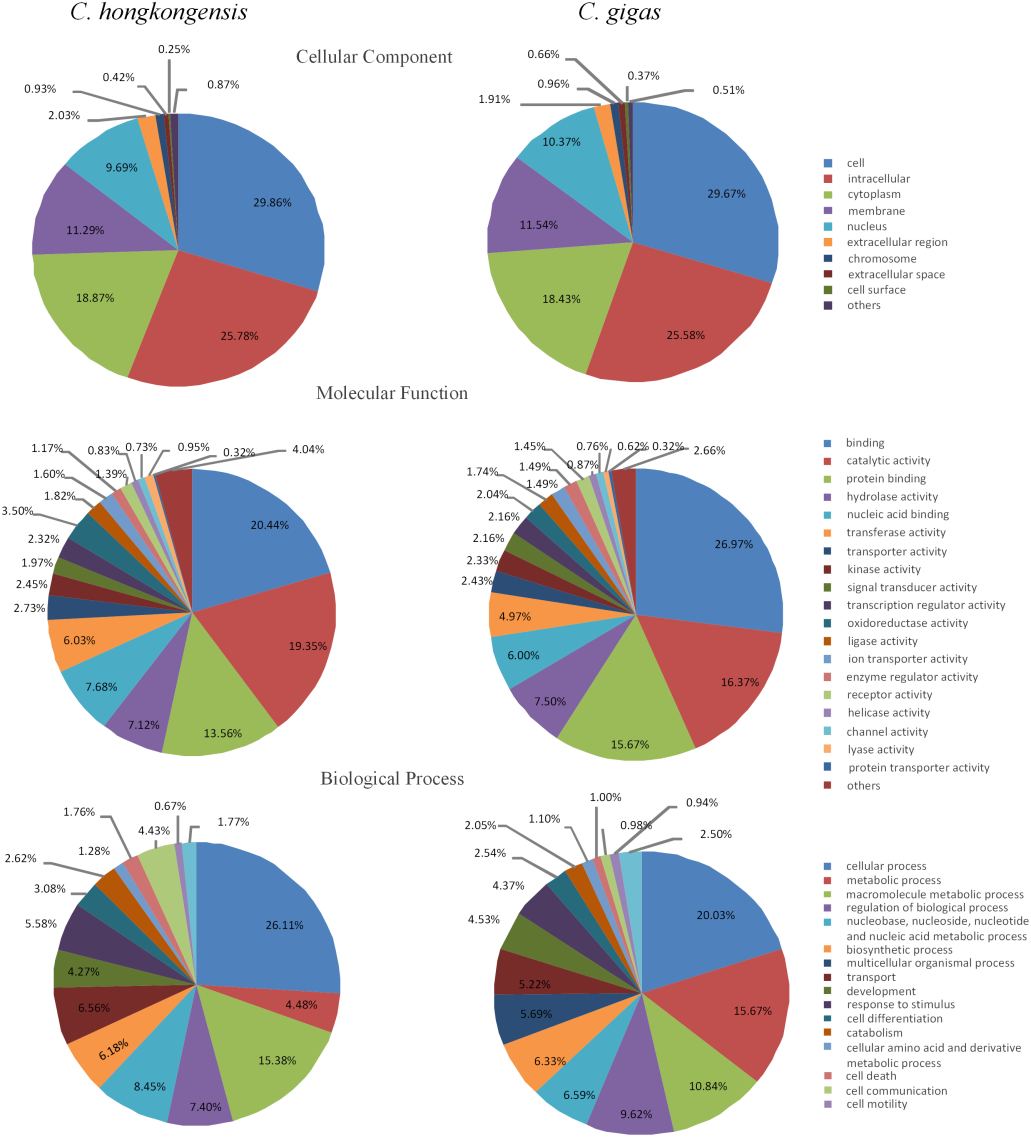
GO comparison between the *C. hongkongensis* and *C. gigas* transcriptome. The *C. gigas* data was from a previous study by Zhao et al. (2012).

Gene Ontology (GO) analysis was widely used to classify gene functions in terms of biological process, molecular function and cellular component [38]. As shown in Figure 2, a total of 9,724 transcripts were assigned with at least one GO term for a total of 57,185 GO assignments. The distribution of assignments of proteins to more specialized GO terms further indicated that *C. hongkongensis* transcripts represent proteins from a diverse range of functional classes (Figure 2). Transport and response to stimulus in biological process may be related to osmotic stress directly. Overall, equal percentages of the transcripts for *C. gigas* and *C. hongkongensis* had GO assignments relating to the three major categories (Figure 2). However, there were some categories that were different between the two species, such as metabolic process and macromolecule metabolic process. This implies that a difference in regulation of metabolic mechanism may exist between the two species in response to low salinity.

**Figure 3:**
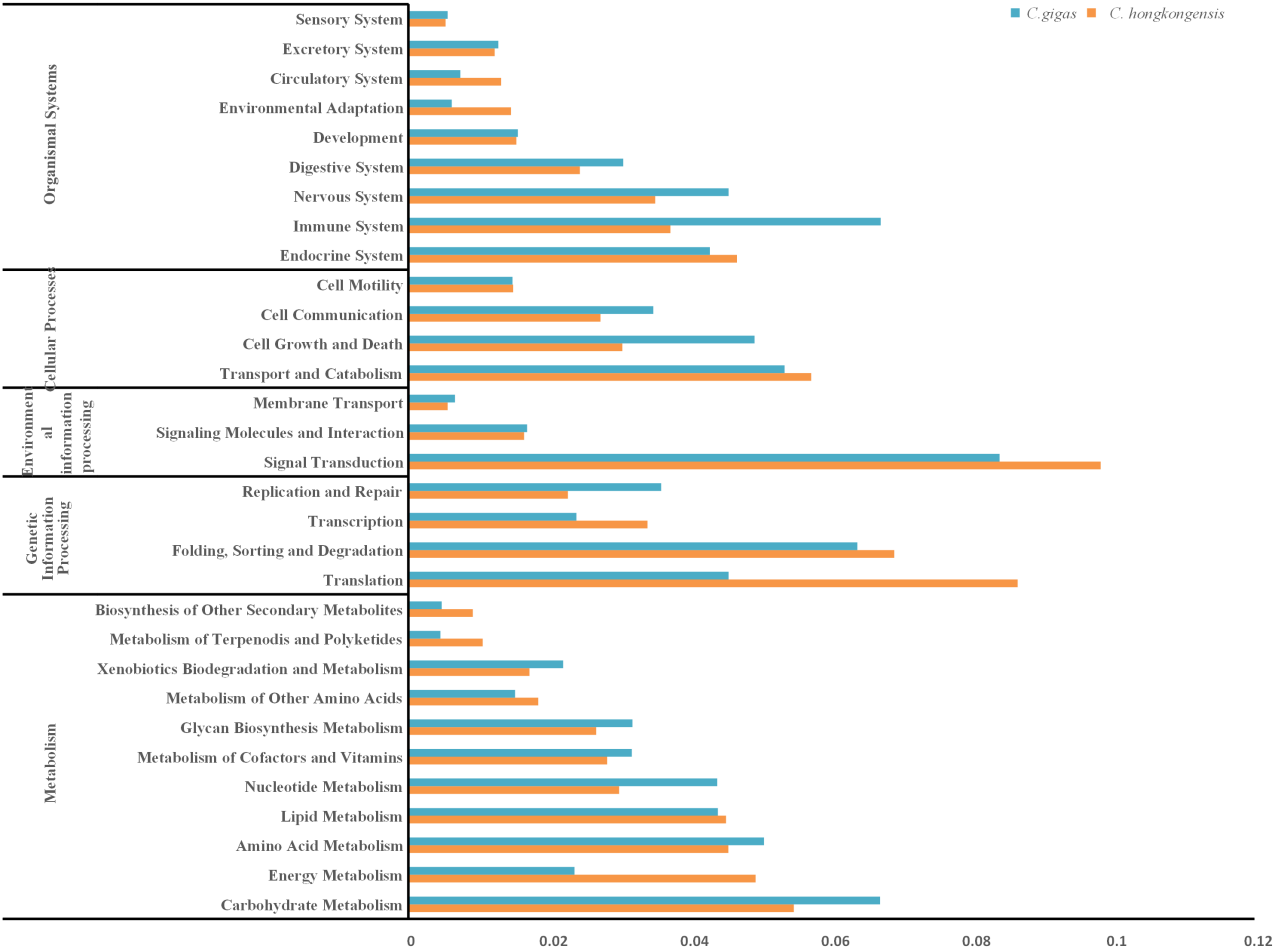
Distribution of the KEGG pathways in both *C. hongkongensis* and *C. gigas*. The bar chart shows the percentages of sequences that are assigned within different pathway categories.

KEGG pathway analysis based on enzyme commission (EC) numbers was performed for all annotated sequences using the KEGG Automatic Annotation Server (KAAS) [39]. The analysis showed that 4,017 sequences were mapped to 273 pathways. The isogroups involved in these pathways are summarized in Figure 3. Signal transduction and translation were two of the well-represented isogroups. With the increasing environmental pressure on the survival of oysters, salinity becomes one of the considerable factors to threaten the living of the oysters. In this KEGG analysis, 12.0% of the isogroups belonged to environmental information processing, which included the most abundant isogroup, signal transduction (634). In addition, several pathways involving immune systems were observed, which are clearly associated with immune response; genes involved in amino acid metabolism may participate in the osmotic regulation were also observed (Figure 3). Comparison of the two oyster species on KEGG pathways showed equal percentages of transcripts assigned to isogroups except immune system, translation and energy metabolism (Figure 3). It might indicate genes related to these three isogroups were the key to adapt to salinity fluctuation. These results will provide a basis for future studies to understand the genetic basis underlying oysters’ adaptation to low salinity and identify gene-associated markers.

### SNP discovery

**Figure 4:**
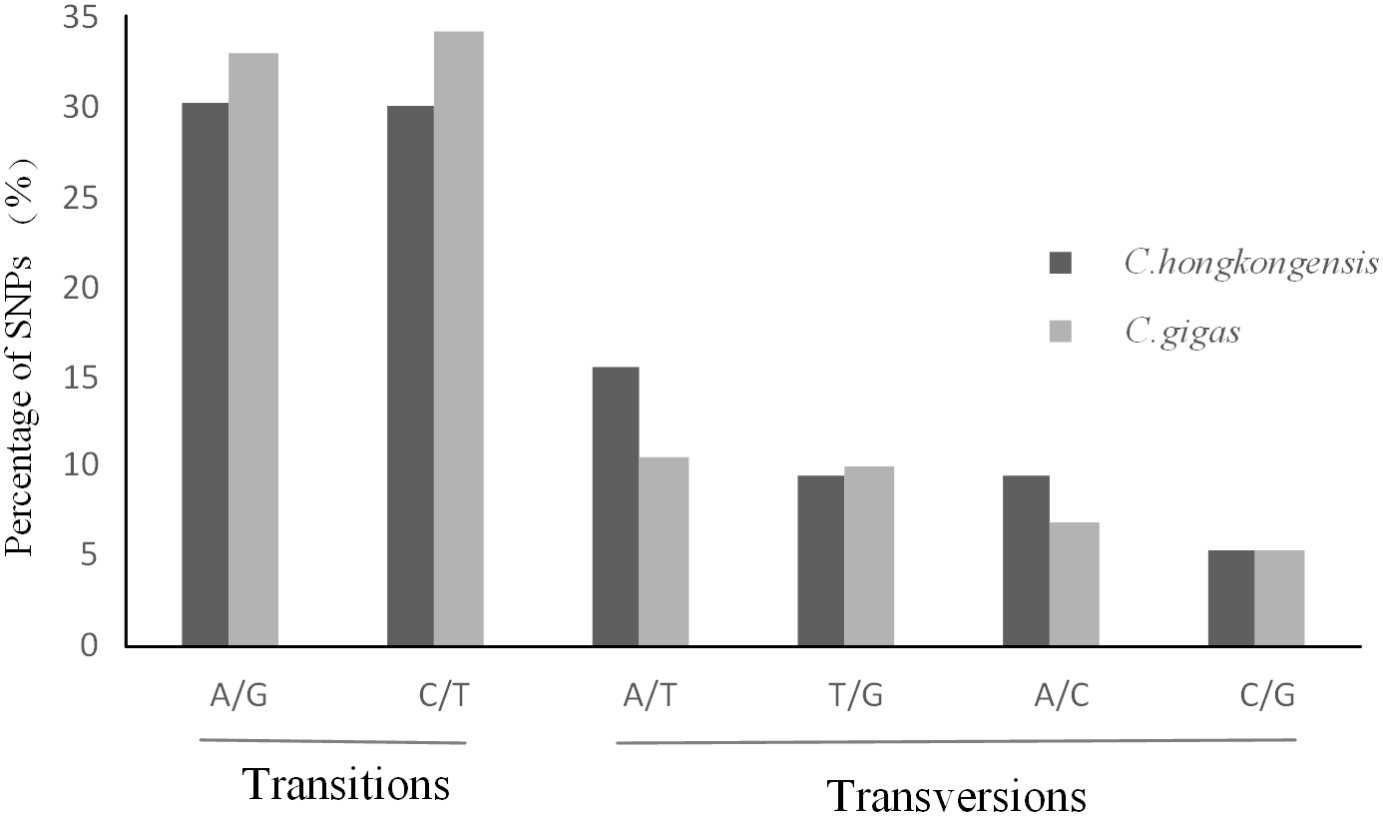
Classification of SNPs identified from the *C. hongkongensis* and *C. gigas* transcriptomes. For both species, transitions occurred more frequently than transerversions. The overall frequency of all types of SNPs was one per 460 bp for *C. hongkongensis* and one per 188 bp for *C. gigas*.

SNPs were identified by variant calling from the alignments generated by the mapping process. After applying several further criteria (see *Material and Methods*), a total of 182,806 putative SNPs were identified from *C. hongkongensis*, and 196,779 putative SNPs were identified from *C. gigas*. For practical application in SNP genotyping assays, only bi-allelic SNPs were considered in this study. The overall frequency of all types of SNPs in the transcriptome of *C. hongkongensis* was one per 460 bp, while that in the transcriptome of *C. gigas* was one per 188 bp. In both species, the distribution of SNP types was similar, with transitions occurring more frequently than transversions (Figure 4). The proportion of transitions in *C. hongkongensis* was lower than that in *C. gigas*, while the transversions were more abundant in *C. hongkongensis*. A/T was the most abundant transversion type and C/G was the least abundant transversion type in both species. These results are similar to those in many other species [22], [36].

Pooling of RNA samples from multiple individuals followed by transcriptome analysis using the next-generation sequencing is one of the most efficient methods for SNP identification [23]. Though *in* *silico* SNP detection allowed for generation of genome-scale SNPs, it is a major problem to minimize the rate of false positive SNPs. The false SNPs might be from paralogous sequence variants (PSVs, single nucleotide differences between duplicated loci in the genome but invariant at the population or species level) or multisite sequence variants (MSVs, single nucleotide variants within duplicated regions) [40]. To minimize the rate of false SNPs, we applied strict criteria to screen quality SNPs in this study. The rate of such errors is expected to decrease with increasing read depth, mapping quality score, and minor allele frequency. We set the minimum read depth of five because this threshold has been shown appropriate to reduce the likelihood of sampling errors [41]. However, SNPs detected within contigs or regions with high sequence depth are more likely to be false SNPs because the sequence reads are more likely from repetitive elements. Therefore, setting a strict minimum minor allele frequency [42] for SNPs detected from larger contigs would reduce the calling of false SNPs [23]. Moreover, in this study, we chose the SNPs detected in both groups to avoiding false SNPs through the technical replication for SNP detection.

In this study, over hundreds of thousands of quality SNPs were generated, for the first time, for marker development in *C. hongkongensis* and *C. gigas*. Validation and testing of SNPs using high-density arrays will be readily applicable relying on the results of this study. A large number of molecular markers are usually required for fine QTL mapping and marker-assisted selection. More specifically, SNP markers occur in protein-coding regions are beneficial for assessing the polymorphisms that directly affect the phenotypes. In addition, the polymorphisms that are associated with adaptive fitness might imply the signatures of natural selection on the genome.

### Identification of putative orthologs

**Figure 5:**
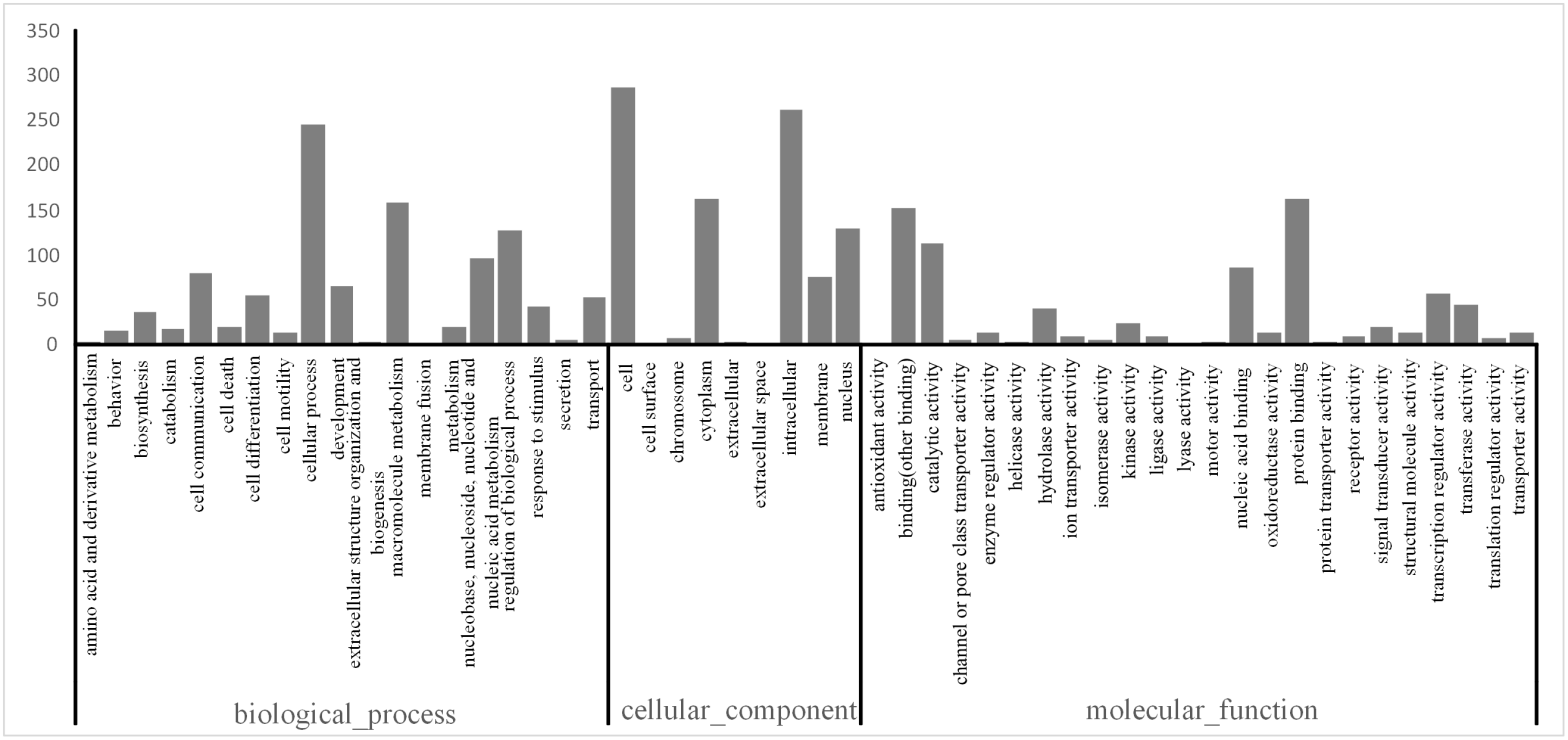
Distribution of GO terms between orthologs of *C. hongkongensis* and *C. gigas.* Function annotation of the 754 ortholog pairs plotted as categories.

We performed blast searches with the predicted CDSs from *C. hongkongensis* against the *C. gigas* genome to identify the putative orthologs between the two species. A total of 11,602 pairs of putative orthologs were identified. After removing the sequences with Ks>0.1 and the sequences with all nonsynonymous substitutions or synonymous substitutions, 754 ortholog pairs were retained for further analysis. The function annotation of the candidate orthologous is showed in Figure 5.

For the past 30 years, protein structure and function were thought to determine the protein evolution exclusively. However, systematic surveys [43] and studies on yeast [44] indicate there are other factors affecting the protein evolution, such as protein expression patterns, genomic position of the encoding genes, their position in biological networks and synonymous codon usage. As shown in Figure 5, the sequence polymorphisms of the 754 orthologous pairs of two *Crassostrea* species are widespread among the GO term categories when using functional annotation. That suggests that protein function was not the chief factor to contribute to the protein evolution. With the development of the advanced sequencing technologies, more systematic analysis would demonstrate the effect of correlated factors.

### Analysis of Ka/Ks

**Figure 6:**
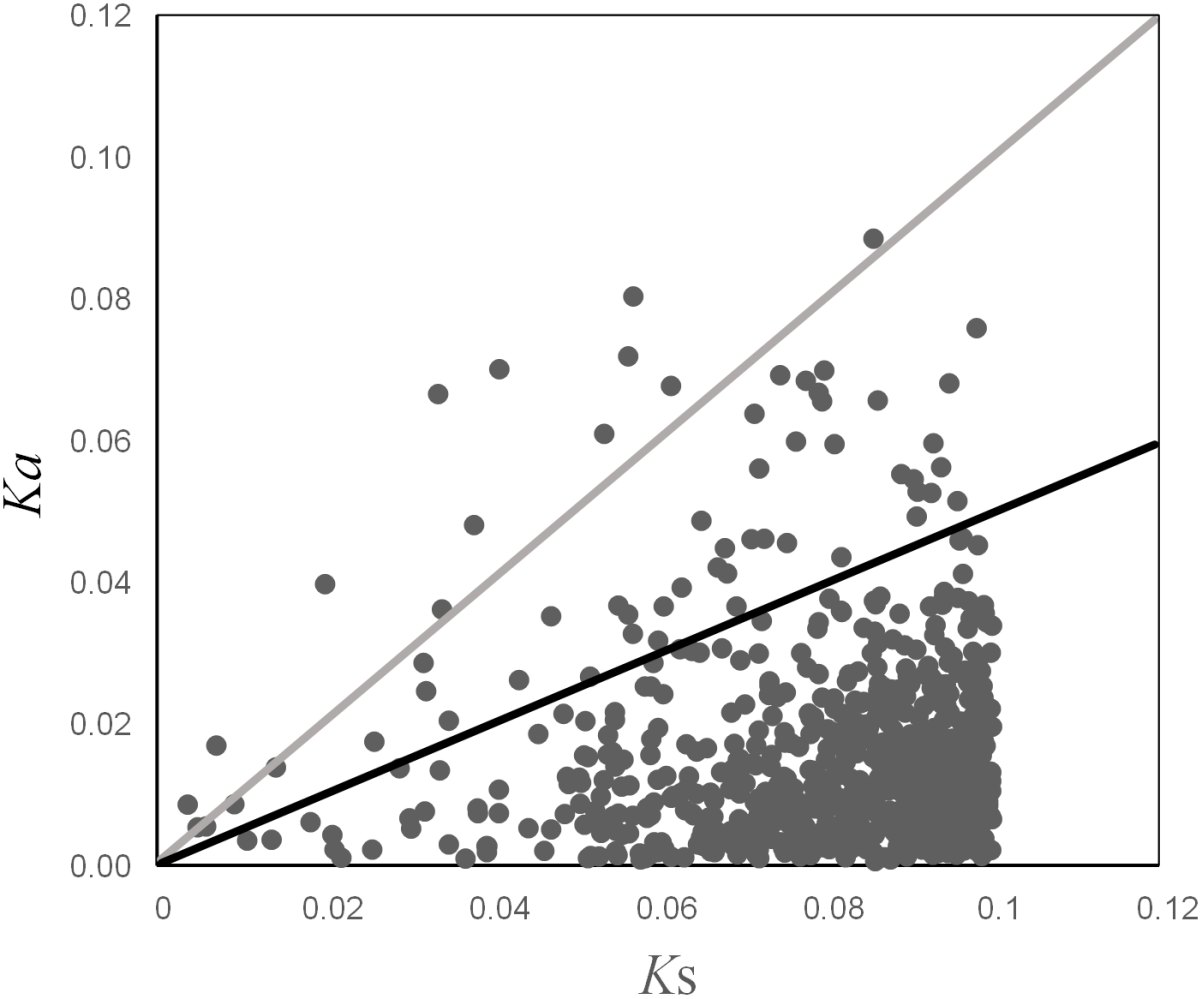
Distribution of Ka/Ks ratio. Ortholog pairs with Ka/Ks ratio >1 are above the grey line, while ortholog pairs with Ka/Ks ratio between 0.5–1 reside between the black and grey lines.

For the data set of 754 unique ortholog pairs that harbored both synonymous and non-synonymous substitutions, the mean values of Ka, Ks and Ka/Ks ratio were 0.0151, 0.0802 and 0.2111, respectively. Of these, 14 ortholog pairs had a Ka/Ks ratio >1, and 45 ortholog pairs had a Ka/Ks ratio between 0.5 and 1 (Figure 6). The genes with Ka/Ks ratio significantly higher than one likely experienced diversifying selection, with which the amino acid change may offer a selective advantage [45]. A Ka/Ks ratio above 0.5 is a less conservative cut-off, but it has also been proven useful for identifying genes under positive selection [46]. Adaptive molecular evolution in most convincing cases have been identified through the Ka/Ks ratio in protein-coding DNA sequences [45]. Therefore, all these 59 orthologs (Ka/Ks>0.5) were considered as candidate genes that have probably experienced positive selection.

### Analysis of candidate genes under positive selection

The detailed information of 59 candidate genes with Ka/Ks between 0.5 and 1 was provided in Table S1. These genes were involved in a variety of functions in zinc ion binding, oxidase activity, metabolic process, immune response and ATP binding. Of the 14 ortholog pairs that had Ka/Ks>1, six genes were annotated and assigned with GO terms (Table 2). Notably, four of these six genes belong to tripartite motif-containing (TRIM) family. TRIM family proteins have been implicated in many biological processes including cell differentiation, apoptosis, transcriptional regulation and signaling pathways [47]. In this study, there are more than one transcripts annotated by *TRIM2*, and these sequences have no similarity among them. Such differences might be related with the need of a better annotation of the oyster genome. In the annotation of oyster genome, some transcripts are annotated with the same annotation because they have the same domain belonging to the annotated protein family and their accurate functions need further researches. TRIM superfamily involves in a broad range of biological processes that are associated with innate immunity [48]. Natural selection often play a role in the evolution of host immune system proteins [49]. In addition, *TRIM2* is reported to regulate cell proliferation in *C. gigas* and respond to pesticides [50], while *TRIM45* acts as a new transcriptional repressor in mitogen-activated protein kinase (MAPK) signaling pathway [51].

**Table 2:** Identification of candidate genes under positive selection (Ka/Ks>1).

Transcript ID	Ka/Ks ratio	Gene name	Gene Ontology
comp104168_c0_seq1 CGI_10007982	1.40	Transient receptorpotential cationchannel subfamilyM member 3	P: GO:0006816 calcium ion transport; P: GO:0055085 transmembrane transport; F: GO:0005262 calcium channel activity; C: GO:0016020 membrane; C: GO:0016021 integral component of membrane
comp26473_c0_seq1 CGI_10001634	1.27	Tripartitemotif-containingprotein 2	P: GO:0016567 protein ubiquitination; F: GO:0008270 - zinc ion binding; F: GO:0004842 ubiquitin-protein ligase activity; F: GO:0005515 protein binding; C: GO:0005622 intracellular; C: GO:0005737 cytoplasm
comp21733_c0_seq1 CGI_10006649	1.23	Tripartitemotif-containingprotein 2	P: GO:0016567 protein ubiquitination; F: GO:0008270 - zinc ion binding; F: GO:0004842 ubiquitin-protein ligase activity; F: GO:0005515 protein binding; C: GO:0005622 intracellular; C: GO:0005737 cytoplasm
comp26605_c0_seq1 CGI_10009487	1.10	Tripartitemotif-containingprotein 2	P: GO:0016567 protein ubiquitination; F: GO:0008270 - zinc ion binding; F: GO:0004842 ubiquitin-protein ligase activity; F: GO:0005515 protein binding; C: GO:0005622 intracellular; C: GO:0005737 cytoplasm
comp15269_c0_seq1 CGI_10015178	1.06	Tripartitemotif-containingprotein 45	F: GO:0008270 zinc ion binding; C: GO:0005622 intracellular; C: GO:0005737 cytoplasm
comp9941_c0_seq1 CGI_10019616	1.03	Transcriptionfactor HES-1-B	P: GO:0000122 negative regulation of transcription from RNA polymerase II promoter; P: GO:0006351 transcription, DNA-templated; P: GO:0007219 Notch signaling pathway; F: GO:0003677 DNA binding; F: GO:0043425 bHLH transcription factor binding; F: GO:0046982 protein heterodimerization activity; C: GO:0005634 nucleus

Only genes with known identities were shown. The full list of genes were provided in Table S1.

The other candidate gene associated with “ion transport”, *transient receptor potential (TRP) cation channel subfamily M member 3*, belongs to TRP superfamily. In many model organisms, it has been shown that TRP proteins are widely distributed and play roles in sensing local changes of stimuli ranging from light to temperature and osmolarity. *TPRM3*, identified in this study, is a Ca^2+^-permeant nonselective channel [52], mediating Ca^2+^ entry into cells. Given the unique importance of Ca^2+^ signaling and its homeostasis in all cell types, TPR channels would play an important role in maintaining the signal transduction and osmotic balance of Ca^2+^. Previous studies report that the Ca^2+^ entry cells increased during treatment with hypotonic extracellular solution [53]. That observation might be related with the salinity adaptation of *C. hongkongensis*.

The transcription factor, *HES1-B*, is also under strong positive selection (Ka/Ks = 1.031). HES1 is a transcriptional repressor and an effector of the notch-signaling pathway that dictates cell fate and critically influences cell proliferation, differentiation, and apoptosis [54]. HES1 is down-regulated in response to hypo-osmotic challenge in gills of killifish and may participate in the remodeling of gill tissue with notch signaling pathway [55]. The transcription factor identified herein may possess the roles in transcriptional regulation of the genes related to hypo-osmotic stress.

### Natural selection between *C. hongkongensis* and *C. gigas*

*C. hongkongensis* has been misidentified as *C. gigas* since the 1970 to 1999 in various studies about this species from Deep Bay, Hong Kong. Lam and Morton verified that *C. hongkongensis* was a genetically distinct taxon by phylogenetic analyses based on the *cytochrome oxidases I* (CoI) and *16S* data [56]. Meanwhile, the phylogenetic analyses suggested a close relationship between *C. hongkongensis* and *C. gigas* [56], [57]. This observation is supported by our results because the vast majority (99.5%) of 11,602 ortholog pairs had Ka/Ks ratios less than 0.5. However, the two species have different inhabitants and should have possessed specific adaptation to environmental factors, such as salinity and temperature. Therefore, these species could provide a model system for understanding the ecology and evolution of adaptive radiations in bivalves.

Owing to the close relationship between the two species, it’s not surprising that only a small number (59) of the genes that experienced positive selection were identified. However, other evolutionary factors may limit the detection of selection signals on genes, i.e. *C. hongkongensis* and *C. gigas* may be mostly weakly diverged at neutral loci. In addition, evolutionarily important changes could occur in the gene regulatory regions rather than the protein-coding regions [58]. For instance, the moderate variations in transcription factors between the species may cause great differences in expression of responsible genes through expression regulation. We found one transcription factor, *HES-1*, under positive selection in our results, future studies on this gene warrant the test of this hypothesis. As shown in our results, natural selection may not play decisive role in salinity adaptation in oyster, so the mechanism needs more researches from different views.

### Genome-wide mutation rate estimate of *Crassostrea* in China

We used interspecific distance estimates based on neutral substitution (Ks) to calculate a transcriptome-wide estimate of substitution rate in *Crassostea* in China. The substitutional mutation rate would be 1.39×10^−9^ per site per year when calibrated to the divergence time about 28.8 Myr ago between the two species [59]. As far as we know, this is the first transcriptome-wide inference of substitution rate for *Crassostrea*. The rate we estimated is considerably slower than the few genome-wide estimated ones, such as the rate of mammals: ∼2.2×10^−9^ per site per year [60] and the rate of humans: ∼3.0×10^−8^ per site per generation [61]. The rate we have generated was just estimated by *in* *silico* and was not an exact value. However, the relative studies in evolution of oyster were few. The result in this study will be reference for researchers of adaptive radiations of mollusca and especially the oyster. It is significative to study the evolution of mollusca which is the second species-rich phylum of the animal kingdom after Arthropoda.

### Differentially expressed genes after osmotic stress

The four groups from the two oyster species were also separately aligned to the each reference transcriptome in order to perform a differential expression analysis between control groups and treated groups of each oyster species. We identified a total of 48 transcripts (pval<0.001) that showed differential expressions between the two groups of *C. gigas* and 408 ones (|log_2_(fold-change)|>1, pval<0.001) between the two groups of *C. hongkongensis*. Among them, there are 15 and 217 transcripts that significantly differentially expressed in *C. gigas* and *C. hongkongensis* with the |log_2_(fold-change)|>1 and padj<0.05 (Table S2, Table S3). The stronger response of mRNAs to low osmotic stress in *C. hongkongensis* than *C. gigas* may related to *C. hongkongensis* being more adaptable. The DEGs contained some important immune-related genes, such as *C-type lectin*, *lysozyme*, *complement C1q protein* and *HSP70* in both two oyster species. There were some genes related to the metabolism of free amino acids, such as *Caspase*, *L asparaginase* and *GMP synthase* that were absent in *C. gigas*. These data provide important information of oyster in osmotic responses. We did not observe any significant correlation between either expression level or differential expression and selective constraint as measured by Ka/Ks. That result is in agreement with what the previous studies found in *Picea abies* [62] and *Pinus halepensis* [63].

### Conclusions

By comparative transcriptome analysis of two oysters, one from seawater and one from brackish water, we identified 14 genes that have experienced strong positive selection (Ka/Ks>1), and 45 genes that may also bear signatures of positive selection (1≥Ka/Ks>0.5). Of these, six genes associated with signaling transduction, ion transport and transcription regulation are likely involved in the adaptive process to hypo-osmotic conditions. Besides, a large set of SNPs were identified, which are expected to provide valuable resources for genetic and evolutionary studies in oysters. The DEGs in response to low salinity stress were also identified in the two oyster species. This study provided the first view of genetic divergence between the two species at the transcriptome level. The genes that display signatures of positive selection will provide the basis for further investigations aiming to understand hypo-osmotic adaptation and species divergence shaped by environmental stress.

## Materials and Methods

### Sample collection

The experiment and sample collection are similar as described in our previous study [27]. Adult individuals of *C. hongkongensis* were collected from Zhanjiang, Guangdong Province, China, in 2010, and were acclimatized for a week in 25‰ filtered seawater at 20°C before experiment. In the experiment, twenty-four oysters were individually tagged and randomly divided into two groups. One was the control group (HC) which was kept in optimal salinity filtered seawater (25‰); the other was the treatment group (HT) which was exposed to the simulated conditions of increased fresh water input (8‰). In order to ensure the free exchange of seawater between the inside and outside of the shell, a part of the shell edge (about 10 mm long and 5 mm wide) of each specimen was chipped away. After 8 hours, six oysters from each group were randomly selected for sample collection. The gill tissues from two groups were dissected and saved in RNA store (Dongsheng Biotech) for RNA extraction, respectively.

### RNA isolation

Total RNA was extracted using the TRIzol reagent according to the manufacturer’s instructions (Invitrogen). The quantity and quality of total RNA were assessed using NanoDrop (Thermo Fisher Scientific) and Agilent 2100 BioAnalyzer (Agilent Technologies). After that, the total RNA was treated with Dnase I (Ambion) following the manufacturer’s protocol. The Poly (A) mRNA was enriched from each total RNA sample using MicroPoly(A)-Purist™ Kit (Ambion) according to manufacturer’s instructions. Equal amount of high-quality mRNA from each individual of the same group were pooled for sequencing.

### High-throughput sequencing

cDNA libraries were prepared following the protocol described in [64]. Briefly, first-strand cDNAs were synthesized using SuperScript II reverse transcriptase (Invitrogen) with an oligo(dT)-adapter primer. Second-strand synthesis was performed with Ex Taq polymerase (Takara). The synthetic cDNA was fragmented to 300–500 bp by a UTR200 sonication device (Hielscher Ultrasonics GmbH), and was purified using AMpure beads (Agencourt). This was followed by amplifying with TruSeq PE cluster kit v3-cBot-HS (Illumina) and constructing libraries with TruSeq™ DNA sample prep kit-set A (Illumina) according to the manufacturer’s instructions. High-throughput sequencing was conducted using Illumina HiSeq™ 2000 platform to generate 100-bp paired-end reads.

### Transcriptome analysis

Raw data generated from Illumina sequencing were trimmed by removing adapter sequences, reads with unknown base calls (Ns) more than 5%, low quality reads (the proportion of low-quality bases (Q<5) more than 50%), and reads with length less than 20 bp. The *de* *novo* transcriptome assembly was carried out with Trinity program, a short read assembler [65]. These high-quality reads have been deposited in the NCBI GEO database with the accession number GSE51157.

The protein-coding sequences were predicted from the assembled sequences by getorf program from EMBOSS package [66]. For the gene annotation, the predicted protein-coding sequences were searched against the *C. gigas* genome, the Swiss-Prot database and the NCBI no-redundant (Nr) protein database using Blastp with an E-value of 1e–5. To increase computational speed, all Blast searches were limited to the top 10 significant hits for each query. Gene identity was assigned to each protein sequence based on the best BLAST hit (with highest bit-score).

For gene ontology analysis, the outputs from the Blastp were imported into GoPipe software [67] to retrieve GO terms at level 2. These GO terms assigned to query protein sequences provide a broad view of genes cataloged with each of the three ontology vocabularies, including biological processes, molecular functions and cellular components. KEGG pathways were analyzed using the online KEGG Automatic Annotation Sever (Kyoto encyclopedia of genes and genomes, http://www.genome.jp/kegg/kaas/) using bi-directional best-hit method (BBH) [13].

### SNP discovery

To identify SNPs from *C. hongkongensis* transcriptome, the clean data from HC and HT groups were mapped against the *de* *novo* assembled transcript sequences using the program BWA [68] with default setting. Similarly, the clean data from *C. gigas* were mapped against the genome of *C. gigas* [15] using the program BWA for identification of SNPs from *C. gigas*. The alignment output from read mapping was then sorted and removed duplicate reads using Picard (http://picard.sourceforge.net/) command line tools. The alignment files were indexed with SAMtools [69] and the dictionary of reference sequences were made using the Picard. The SNP calling was performed with the Genome Analysis Toolkit (GATK 2.7) [70]. High-quality variants were called with Unified Genotyper using a minimum Phred quality score of 30 [71]. The identified SNPs were further filtered using the following criteria: (1) the SNPs were discovered from both groups; (2) the read coverage was at least 5 reads; (3) the minor allele frequency was at least 20%.

### Identification of orthologs

We used the bidirectional best hit method to identify putative orthologs between the two species using tblastx with the bit-score threshold of 300 [46]. To avoid the inclusion of paralogs, we only retained those ortholog pairs that matched the same proteins by BlastX searches. Coding sequences (CDSs) of the filtered orthologous gene pairs were determined based on the prediction output of getorf as mentioned above after removal of CDSs with unexpected stop codons. The filtered CDSs of orthologous gene pairs were then aligned by ClustalW 2.1 [72] for downstream analysis.

### Test for positive selections

The ratio of the number of nonsynonymous substitutions per nonsynonymous site (Ka) to the number of synonymous substitutions per synonymous site (Ks) was used to test for positive selection. We estimated the rate of Ka to Ks between putatively orthologous coding regions based on the maximum-likelihood method [73] using KaKs_Calculator 2.0 with the YN model [74]. The orthologs with a Ks rate >0.1 were excluded from further analysis to avoid inclusion of paralogs [46].

### Estimating the overall substitutional mutation rate

We estimated an overall substitutional mutation rate for the *Crassostrea* based on divergence between orthologous pairs and synonymous mutations calibrated with the estimated divergence time [59]. The rate (*r*) (in substitutions/site/year) is calculated from the mean genetic distance (*d*) between two species (*2t*), while *d* for coding regions is based on the Ks rate [46].

### Differentially expressed genes

The unigene expression was calculated using RSEM [75]. Then the DESeq R package [76] was employed to identify up-regulated and down-regulated genes between the control and treated groups of both two oyster species. As only two groups of each species were used in analysis of differential expression (no biological replicates), results have to be interpreted with caution. Normalization was made using size factors after calculation of relative library sizes as manual suggestion.

## Supporting Information

Table S1 Putative orthologous genes with Ka/Ks>0.5.
(XLSX)

Table S2 Differentially expressed genes with annotation of *Crassostrea hongkongensis*. HT: genes were only found in the HT group; HC: genes were only found in the HC group; *: padj>0.05.
(XLSX)

Table S3 Differentially expressed genes with annotation of *Crassostrea gigas*. PT: genes were only found in the PT group; PC: genes were only found in the PC group; *: padj>0.05.
(XLSX)
