# Correction: Comparative Transcriptome Analysis of Two Oysters, *Crassostrea gigas* and *Crassostrea hongkongensis* Provides Insights into Adaptation to Hypo-Osmotic Conditions

**DOI:** 10.1371/journal.pone.0118665

**Published:** 2015-03-05

**Authors:** The PLOS ONE Staff

There are errors in the annotation column of S2 Table. Please view the correct table, renamed S2 Table, below.

## Supporting Information

S2 Table Differentially expressed genes with annotation of *Crassostrea hongkongensis*. HT: genes were only found in the HT group; HC: genes were only found in the HC group; *: padj>0.05.
(XLSX)
